# Cytocompatible and Anti-bacterial Adhesion Nanotextured Titanium Oxide Layer on Titanium Surfaces for Dental and Orthopedic Implants

**DOI:** 10.3389/fbioe.2019.00103

**Published:** 2019-05-09

**Authors:** Sara Ferraris, Andrea Cochis, Martina Cazzola, Mauro Tortello, Alessandro Scalia, Silvia Spriano, Lia Rimondini

**Affiliations:** ^1^Department of Applied Science and Technology, Politecnico di Torino, Turin, Italy; ^2^Department of Health Sciences, Università del Piemonte Orientale “UPO”, Novara, Italy; ^3^Interdisciplinary Research Center of Autoimmune Diseases, Center for Translational Research on Autoimmune and Allergic Diseases–CAAD, Novara, Italy

**Keywords:** nanotexture, titanium, bone contact, bacterial adhesion, surface modification

## Abstract

It is widely recognized that surface nanotextures applied on a biomaterial can affect wettability, protein absorption and cellular and/or bacterial adhesion; accordingly, they are nowadays of great interest to promote fast osseointegration and to maintain physiological healing around biomedical implants. In order to be suitable for clinical applications, surface nanotextures must be not only safe and effective, but also, they should be produced through industrial processes scalable to real devices with sustainable processes and costs: this is often a barrier to the market entry. Based on these premises, a chemical surface treatment designed for titanium and its alloys able to produce an oxide layer with a peculiar sponge like nanotexture coupled with high density of hydroxyl group is here presented. The modified Ti-based surfaces previously showed inorganic bioactivity intended as the ability to induce apatite precipitation in simulated body fluid. Physicochemical properties and morphology of the obtained layers have been characterized by means of FESEM, XPS, and Zeta-potential. Biological response to osteoblasts progenitors and bacteria has been tested. The here proposed nanotextured surfaces successfully supported osteoblasts progenitors' adhesion, proliferation and extracellular matrix deposition thus demonstrating good biocompatibility. Moreover, the nanotexture was able to significantly reduce bacteria surface colonization when the orthopedic and the periodontal pathogens *Staphylococcus aureus* and *Aggregatibacter actinomycetemcomitans* strains were applied for a short time. Finally, the applicability of the proposed surface treatment to real biomedical devices (a 3D acetabular cup, a dental screw and a micro-sphered laryngeal implant) has been here demonstrated.

## Introduction

Titanium and its alloys (mainly Ti6Al4V) are the most widely employed materials for orthopedic and dental implants due to their good mechanical properties and biocompatibility. However, crucial improvement of their bone integration ability and reduction of bacterial contamination is still a challenge. Moreover, it should be considered that Ti-based materials implantation involves also a contact with soft tissues (e.g., dental implant collar, percutaneous implants, laryngeal implants): so, a new field of investigation has been recently developed to focus on this type of material-tissue integration (Linkevicius and Vaitelis, 2015; Salvi et al., 2015; Ferraris et al., 2017a,b).

Several strategies have been proposed in the scientific literature, in patents and in clinical applications to improve bone bonding ability of titanium surfaces or other bone substitutes such as bioglass (Vernè et al., 2014) and stainless steel 316L (Le et al., 2013). Modification of surface topography, bioactive coatings and chemical/electrochemical treatments aimed at obtaining bioactive oxide layers can be cited as the most important ones (de Jonge et al., 2008). These strategies are based on the consolidated knowledge that surface topography, chemical composition and charge are the main factors affecting tissue-surface biological interactions. Thus, an increasing interest on biological functionalization (surface grafting of specific biological molecules) has also been registered in the last years to improve the bone-implant interaction (Bhola et al., 2011). On the opposite, despite a significant increase in interest, surface modifications aimed at the improvement of the interactions with soft tissues are still less investigated (Ferraris et al., 2017a,b).

Topography has a crucial role in osseointegration and it is well-known that cell response can be modulated by tailoring the surface texture of the implant. A complex topography with the simultaneous presence of roughness on the micro, sub-micro and nano scale can be very effective in promoting osseointegration (Gittens et al., 2011). In general, the micro and sub-micro roughness (Ra = 0.4–2 μm), with dimension comparable with the cells size, enhances cell differentiation and release of local factors involved in the osseointegration. On the opposite, nanoscale roughness with size comparable with the one of proteins and cell membrane receptors could improve cell adhesion and spreading. In fact, it has been shown that the presence of roughness at the micro-scale can be successful in promoting osteoblasts differentiation (Gittens et al., 2011); however, to advance osteoblasts fast proliferation, a secondary nano-scale structure was required (Gittens et al., 2011). Accordingly, to successfully stimulate osseointegration, a combination of micro- and nano-complex topography appears as the most promising combination of topographical stimulation (Gittens et al., 2012, 2013). The principal approaches developed and used to reach the appropriate roughness on implants are grit blasting, sand blasting, acid etching, anodization, and titanium plasma spray (Le Guéhennec et al., 2007). Other proposed strategies to improve osseointegration of the titanium surfaces were performed with coatings of hydroxyapatite (Sul and Towse, 2014), bioactive glasses (Lopez-Sastre et al., 1998), bisphosphonates (Lee et al., 2011), and collagen (Sverzut et al., 2012).

The problem of prosthetic infections and the concomitant development of increasing bacterial resistance to common antibiotics are at the basis of a growing attention to antibacterial or antiadhesive surfaces for medical implants (Zhao et al., 2009; Simchi et al., 2011). The rationale of these surfaces includes both anti-adhesive materials (able to reduce bacterial adhesion to their surface) and active antibacterial materials (which can exert a bactericidal activity).

According to these premises, in the last years, the attention of scientists has been focused on surface features on the nanoscale aimed to both improving cells-surface interactions and reducing bacterial colonization using bioactive compounds (e.g., silver nanoparticles) (Rai et al., 2009; Variola et al., 2011). The choice between an anti-adhesive or active antibacterial surface is highly debated. On one hand, the introduction of an active antibacterial agent (drug or metal ions/nanoparticles) changes the device classification for Conformité Européene (CE) or Food and Drug Administration (FDA) marking and makes the certification *iter* much more complex. On the other hand, a surface able to actively counteract the infection is much more effective when the infection risk is high. Another source of debate is the use of antibiotics, inorganic or alternative antibacterial agents (e.g., antimicrobial peptides, chitosan, etc.). Antibiotics are well-accepted and are almost the only antibacterial agents introduced into commercial biomedical implants until now; however, they are limited by increasing bacterial resistance and even if they can effectively withstand a specific infection, they can hardly face a polymicrobial progression due to the protection that each single strain offers to others (de Vos et al., 2017). Inorganic antibacterial agents (e.g., silver) have a broad spectrum of activity (they can prevent contamination from unknown bacteria and act against polymicrobial infections) but have a shorter clinical history; moreover, it is not so easy to find the optimal therapeutic window to obtain antibacterial activity without introducing cytotoxicity toward host tissues (Brouse et al., 2017).

In this work, as an alternative to the prior debated techniques, an innovative surface chemical treatment for Ti-based biomedical implants is described: it allows to get a nanotextured oxide layer rich in hydroxyl groups and it can be applied to get a complex surface topography of an implant. Of course, there are several well-assessed etching procedures to successfully introduce micro-roughness onto titanium implants. However, the here proposed process allows for the development of a peculiar nanotextured surface that can be overlapped to various micro- or macro-surface topographies; moreover, it allows to complete the entire treatment in a single step (based on controlled oxidation in hydrogen peroxide), thus avoiding the use of expensive equipment and reagents. So, in comparison with most of the complex and time-consuming techniques proposed in the literature, this easy, fast and economic method seems to be much more scalable to the industrial needs for the mass production of titanium-based medical devices.

Accordingly, to confirm the use of this treatment for biomedical devices in contact both with bone and soft tissues, an acetabular cup, a dental implant screw and samples of sintered titanium micro-spheres representative of laryngeal implants were used as substrates for the treatment and the main results of their physical-chemical characterization are here reported. Finally, cytocompatibility has been verified toward human osteoblast progenitors as well as the ability of the treated surface to reduce bacterial adhesion has been tested toward the orthopedic pathogen *Staphylococcus aureus* strain and the periodontal infection related strain *Aggregatibacter actinomycetemcomitans*.

## Materials and Methods

### Samples Preparation

Ti6Al4V discs (10 mm diameter, 2 mm thickness) were obtained from cylindrical bars (Titanium Consulting and Trading, Buccinasco, Italy) by automatic cutting. Samples were then polished by SiC abrasive papers (up to 4,000 grit) on an automatic mechanical polishing machine and washed in an ultrasonic bath 5 min in acetone and then 10 min (twice) in ultrapure water (MilliQ, Millipore, MA, USA). The quality of the surface was verified by optical microscopy in order to guarantee specimens' homogeneity. These samples will be named Ti6Al4V–MP (mirror polished) from now on. Specimens' surface was further modified as prior described (Ferraris et al., 2011a) and patented (Spriano et al., EP2214732) by the Authors: the treatment foresees a first etching in diluted hydrofluoric acid (Sigma-Aldrich, Milan, Italy) followed by a controlled oxidation in hydrogen peroxide (Sigma-Aldrich, Milan, Italy). These samples will be named Ti6Al4V–CT (chemically-treated) from now on.

An (i) acetabular cup produced through selective electron beam melting by Arcam EBM (Arcam EB, Mölndal, Sweden), a (ii) dental implant screw treated by a dual etching process (ZIRTI-Sweden&Martina, Padova, Italy), and (iii) samples of sintered titanium micro-spheres, representative of laryngeal implants (Protip Medical, Strasbourg, France) were also used as substrates for the CT treatment. The proposed treatment was directly applied on small samples obtained from the commercial acetabular cup and on samples of sintered titanium microspheres, while it was integrated with an industrial dual acid etching treatment for the dental implant, as previously described by the Authors (Ferraris et al., 2015).

### Chemical and Physical Characterization

Surface morphology of the samples, as well as semi-quantitative chemical composition, was investigated by means of Field emission Scanning Electron Microscopy equipped with energy Dispersive Spectroscopy (FESEM-EDS, MERLIN, Carl Zeiss, Oberkochen, Germany).

The surface of the CT samples was characterized by means of Force atomic microscope observation in tapping mode by means of the Bruker Innova® Atomic Force Microscope with Bruker AFM silicon probe (model RTESPA-CP). The sample CT was put on the holder of the microscope and the tip moved with a 350 kHz frequency and a 3.24 V force. Scans of 5, 1, and 0.5 μm portions of the samples were performed with a resolution of about 10 nm. For the selected areas the values of Average Roughness (Sa) and Root Mean Square Roughness (Sq) were obtained. This analysis was performed in order to investigate the topography of the CT samples.

### Biological Characterizations

Specimens were sterilized by ethanol (eth, 70% in ddH2O) immersion. Briefly, discs were seeded onto a 12 multiwell plate and submerged with 1 ml/each of 70% eth for 2 h at room temperature; then, specimens were carefully washed 3 times with sterile PBS (1 ml/each from Sigma-Aldrich, Milan, Italy) and transported to a new multiwell plate. Plate was stored at room temperature protected from light with aluminum foil until experiments.

#### *In vitro* Cytotoxicity Evaluation

##### Cells

Human osteoblasts progenitor cells hFOB 1.19 were used to assay specimens' cytocompatibility. Cells were purchased from the American Type Culture Collection (ATCC, VA, USA, CRL11372) and cultivated using a Dulbecco's modified eagle's (DMEM)/F12 mix (DMEM/F12, 50:50, Sigma-Aldrich, Milan, Italy) supplemented with 10% fetal bovine serum (FBS, Lonza, Milan, Italy), 1% antibiotics (penicillin/streptomycin) and 3mg/ml neomycin (G418 salt, Sigma-Aldrich, Milan, Italy) at 34°C, 5% CO_2_. When cells reached 80-90% confluence, they were detached by trypsin/EDTA solution, collected and used for experiments. Cells were used until passage 10 to ensure purity.

##### Direct cytocompatibility evaluation

Sterile specimens were gently seeded to a new 12 multiwell plated by sterile tweezers avoiding any surface damage. Then, a defined number of cells (1 × 10^4^ cells/specimens) were dropwise (100 μl) seeded directly onto specimens' surface and allowed to adhere for 2 h at 34°C, 5% CO_2_. Afterwards, each well was rinsed with 1 ml of fresh medium and the cells were cultivated for 1–2–3 days. At each time-point, specimens were firstly moved to a new multiwell plate and then cells viability was verified by the metabolic colorimetric alamar blue assay (alamarBlue®, Thermo Fisher, Waltham, MA, USA) following the manufacturer's instructions. Briefly, at each time-point, supernatants were removed from each well containing cells and replaced with alamar blue solution (10% v/v in fresh medium). Plates were incubated in the dark for 4 h and then 100 μl were removed, spotted into a new 96-well plate and fluorescence signals were evaluated with a spectrophotometer (Victor, Perkin Elmer, Waltham, MA, USA) using the following set-up according to the Manufacturer's instructions: fluorescence excitation wavelength 570, fluorescence emission reading 590 nm. As a control, Alamar™ solution in contact with test materials solely (intended as cells-free) was applied and compared with the fluorescence of the same solution to exclude any reading background due to the reactive groups on the surface.

##### Morphology

After 3 days in culture the morphology of seeded cells was visually checked by immunofluorescent imaging (IF). For IF staining, cells were fixed at room temperature by Immunofix solution (Bio Optica, Milan, Italy) for 15 min; then, they were washed 3 times with PBS and stained for collagen deposition using an anti-collagen I antibody (ab34710, from AbCam, Cambridge, UK) overnight at 4°C. The day after, collagen was unmasked by an appropriate secondary antibody and cells were co-stained with phalloidin (ab176759, AbCam, Cambridge, UK) and 4′,6-diamidino-2-phenylindole (DAPI, Sigma-Aldrich, Milan, Italy) to visualize cytoskeleton f-actin filaments and nuclei, respectively.

#### Evaluation of Biofilm Formation

##### Strains and growth conditions

The pathogen, strong biofilm formers, multi-drug resistant strains *Staphylococcus aureus* (*S. aureus*, ATCC 43300) and *Aggregatibacter actinomycetemcomitans* (*A. actinomycetemcomitans*, ATCC 33384) were used to test specimens' antiadhesive properties as representative for orthopedic and periodontal infections, respectively.

Strains were purchased from the American Type Culture Collection (ATCC, VA, USA); *S. aureus* was cultivated in Trypticase Soy Agar (TSA, Sigma-Aldrich, Milan, Italy) while *A. actinomycetemcomitans* was cultivated in Blood agar plates (Sintak, Milan, Italy). Bacteria were incubated at 37°C until round single colonies were formed; then, 2–3 colonies were collected and spotted into 30 ml of Luria Bertani broth (LB, Sigma-Aldrich, Milan, Italy). Broth cultures were incubated overnight at 37°C in agitation (120 rpm in an orbital shaker), then bacteria concentration was adjusted until 1 × 10^5^ cells/ml by diluting in fresh media until optical density of 0.001 at 600 nm was reached as determined by spectrophotometer (Victor, Packard Bell, LA, USA).

##### Biofilm formation

Sterile specimens were gently located into a 12 multiwell plate by sterile tweezers avoiding any surface damages. Each specimen was submerged with 1 ml of the broth bacteria culture prepared as described in Strains and growth conditions plate was incubated for 90 min in agitation (120 rpm) at 37°C to allows the separation between adherent biofilm cells and not-adherent floating planktonic cells (separation phase). Afterwards, supernatants containing planktonic cells were removed and replaced with 1 ml of fresh media to cultivate surface-adhered biofilm cells (growth phase) (Harrison et al., 2010). Biofilm were grown at 37°C for 24 h prior to evaluations.

##### Biofilm metabolic activity

At the selected time-point, specimens were collected, washed carefully 3 times with sterile PBS to remove non-adherent cells and seeded into a new plate. Then, surface adhered biofilm metabolic activity was evaluated by the metabolic colorimetric 2,3-bis-(2-methoxy-4-nitro-5-sulphenyl)-(2H)-tetrazolium-5-carboxanilide (XTT, Sigma-Aldrich, Milan, Italy). Briefly, 100 μl of the XTT solution (3 mg/ml in PBS supplemented with 1 mmol/l menadione) were added to each well; then, plate was incubated at 34°C, 5% CO_2_ in the dark for 5 h. Afterwards, 100 μl were collected from each supernatant, spotted into a 96 multiwell plate and the optical density evaluated by a spectrophotometer (Victor, Packard Bell, LA, USA) using a 490 nm wavelength.

##### Colony forming units count

After the metabolic assay, a colony forming unit (CFU) count was performed as previously described by Harrison et al. (2010). Specimens were directly infected by a defined bacterial solution as described in Strains and growth conditions paragraph. After 24 h of direct contact, specimens were moved to tubes containing 1 ml of PBS and the biofilm was detached from specimens by sonicator and vortex (30 s, 3 times each); this protocol has been demonstrated as the most effective in detaching bacterial biofilm from different type of surfaces, including rough and porous ones (Schmidlin et al., 2013; Furustrand et al., 2015) in order to ensure a correct CFU count. Then, 100 μL of supernatant were collected from each well and used to perform six serial 10-fold dilutions, mixing 20 μL of bacterial suspension with 180 μL of sterile PBS. Twenty microliter were then collected from each dilution, spotted onto plates containing LB agar medium, and incubated for 24 h at 37°C. Lastly, the CFU mL^−1^ were counted as follows:

*CFU* = *[(number of colonies x dilution factor)*^∧(*serialdilution*)^*]*

where:

number of colonies = countable single round colonies;

dilution factor = dilution made from the initial 1 mL suspension;

serial dilution = 1–6 10-fold dilution areas where colonies were counted.

##### Scanning electron microscopy

To verify successful bacterial detachment and ensure a correct colony counting, specimen's surface was visually checked before and after applying detachment procedures (above detailed in Colony Forming Units count). Briefly, specimens were fixed overnight in 4% glutaraldehyde (from Sigma-Aldrich, Milan, Italy, 4°C, diluted in cacodylate buffer) and then dehydrated by alcohol scale (50-70-90-100%, 2 h each). Then, samples were treated in hexamethyldisilazane (Sigma-Aldrich, Milan, Italy) for 20 min at room temperature, mounted onto aluminum stubs with conductive carbon tape to undergo surface metallization by means of a chromium layer and observed with a FESEM-EDS MERLIN using secondary electrons (Carl Zeiss, Oberkochen, Germany).

### Statistical Analysis of Data

All experiments were performed in triplicate. Data were analyzed using SPSS software (v25, IBM, NY, USA) by means of one-way ANOVA followed by the Sheffé's test as *post-hoc* analysis. Significance level was set at *p* < 0.05.

## Results and Discussion

### Chemical and Physical Characterization of the Flat Model Specimens

**Figure 1:**
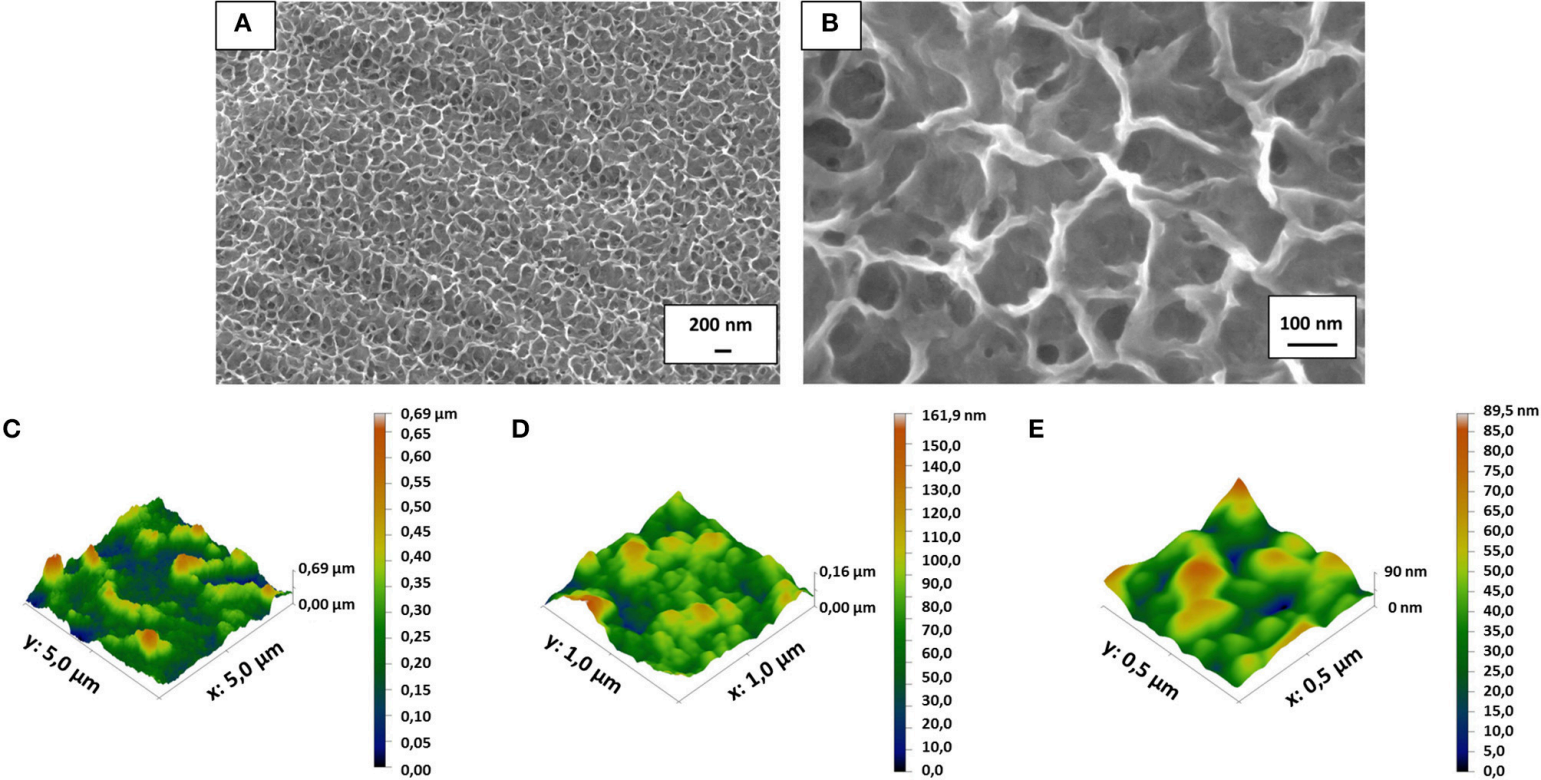
FESEM images revealed the presence of the nanotextured oxide layer obtained after the chemical treatment on the mirror polished flat Ti6Al4V discs **(A,B)**. Surface roughness obtained by AFM measurements **(C–E)** confirmed that the surface texture is in the nanoscale (10–100 nm depending on the measurement area due to the complex roughness at the nano- and micro-scale of the surface).

After mirror polishing, the surface of Ti6Al4V discs was treated to obtain flat model specimens. The obtained results were similar to those previously shown by the Authors (Ferraris et al., 2011a, 2018). As it is displayed in Figure 1, a uniform and homogeneous sponge-like nanotextured oxide layer was obtained after the chemical treatment (a, b). AFM analyses confirmed the presence of the sponge-like nanotexture (Figures 1C–E). Figures 1B,E correspond to the appearance of the treated surface, respectively at SEM and AFM with comparable magnification: in both cases the surface appears as porous with sockets and protruding areas with lateral dimension in the range of 100–250 nm. The Average Roughness (Sa) and Root Mean Square Roughness (Sq) of the CT samples depend on the size of the analyzed area: they were respectively 77.7 and 100.7 nm on an area of 5 × 5 μm, 15.9 nm and 20.6 nm on an area of 1 × 1μm and 10.5 and 13.2 nm on an area 0.5 × 0.5 μm. The roughness decreases with decreasing the size of the analyzed areas because the sponge- like topography of the surface on the nano scale is overlapped to a topography with a small roughness on the microscale (due to the presence of the alpha and beta crystalline phases in the Ti6Al4V alloys which are differently etched) (Gammon et al., 2004) which has much more influence on the measurement as larger is the size of the considered area. This means that the effective roughness of the sponge like topography is around 10 nm. This type of roughness is suitable for bone contact application and can enhance the adhesion of eventual coatings applicable to the surface.

XPS analysis, previously reported by the authors on CT samples (Ferraris et al., 2011a, 2018), evidenced a significant increase in the amount of surface hydroxyl groups on the surface of the treated sample (Ti6Al4V-CT) in comparison with the untreated mirror polished ones (Ti6Al4V-MP), as it can be deduced from the ratio of the Ti-OH and Ti-O peaks. Even if this ratio is not perfectly constant on different samples, it is at least 1:2 on all the treated samples.

Zeta potential measurements, performed by means of the streaming potential technique on solid samples were previously reported by the authors in (Ferraris et al., 2018) for CT and MP surfaces, confirmed the presence on the treated samples of a surface layer rich in a single type of chemical group with acidic behavior, as evidenced by a plateau in the basic region. The presence of chemical groups with acidic behavior was confirmed by the shift in the isoelectric point (IEP) from 4.7 (before the treatment) to ≈ 2 observed after the chemical treatment (Ferraris et al., 2018). The onset of the plateau at low pH (around 4) demonstrated that all the OH groups exposed on the surface act as a strong acid. According to the different surface functional groups, the zeta potential of the two surfaces at physiological pH was significantly different: both have a negative surface charge that is due to deprotonated OH groups in the case of the Ti6Al4V-CT sample and to adsorbed OH groups from the solution in the case of the Ti6Al4V-MP sample (Ferraris et al., 2018).

The presence of OH groups is an important feature: in fact, it is strongly related to surface wettability, bioactivity and protein adsorption, as well as it can affect coating adhesion (Ferraris et al., 2011a, 2018). The negative charge of the surface due to complete deprotonation of the OH group at physiological pH not only strongly attracts the water biomolecules (higher wettability) and ions (Ca^2+^, ${\text{PO}}_{4}^{3-}$) in the physiological fluids (bioactivity with precipitation of hydroxyapatite), but also allows for a stronger electrostatic interaction with all the proteins which are far from their IEP at physiological pH (e.g., albumin and fibronectin) and enhances the adhesion of an eventual coating deposited on the surface.

### Chemical and Physical Characterization of the Surface Modified Biomedical Devices

**Figure 2:**
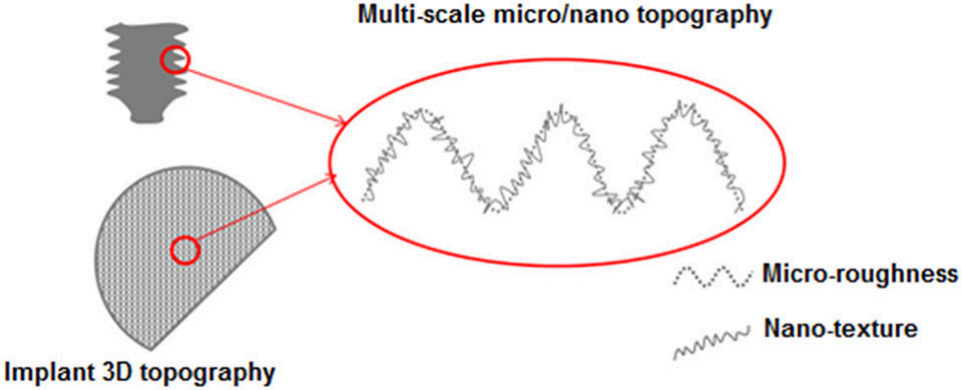
Schematic representation of the multi-scale surface topography.

A complex topography can be obtained by applying the described surface chemical treatment to a biomedical device with a surface profile with roughness on the macro or micro-scale, differently tailored according to the different devices. The sponge-like nanotexture can be overlapped to micro or macro topographies in order to obtain a fully multi-scale roughness/topography, as schematized in Figure 2.

**Figure 3:**
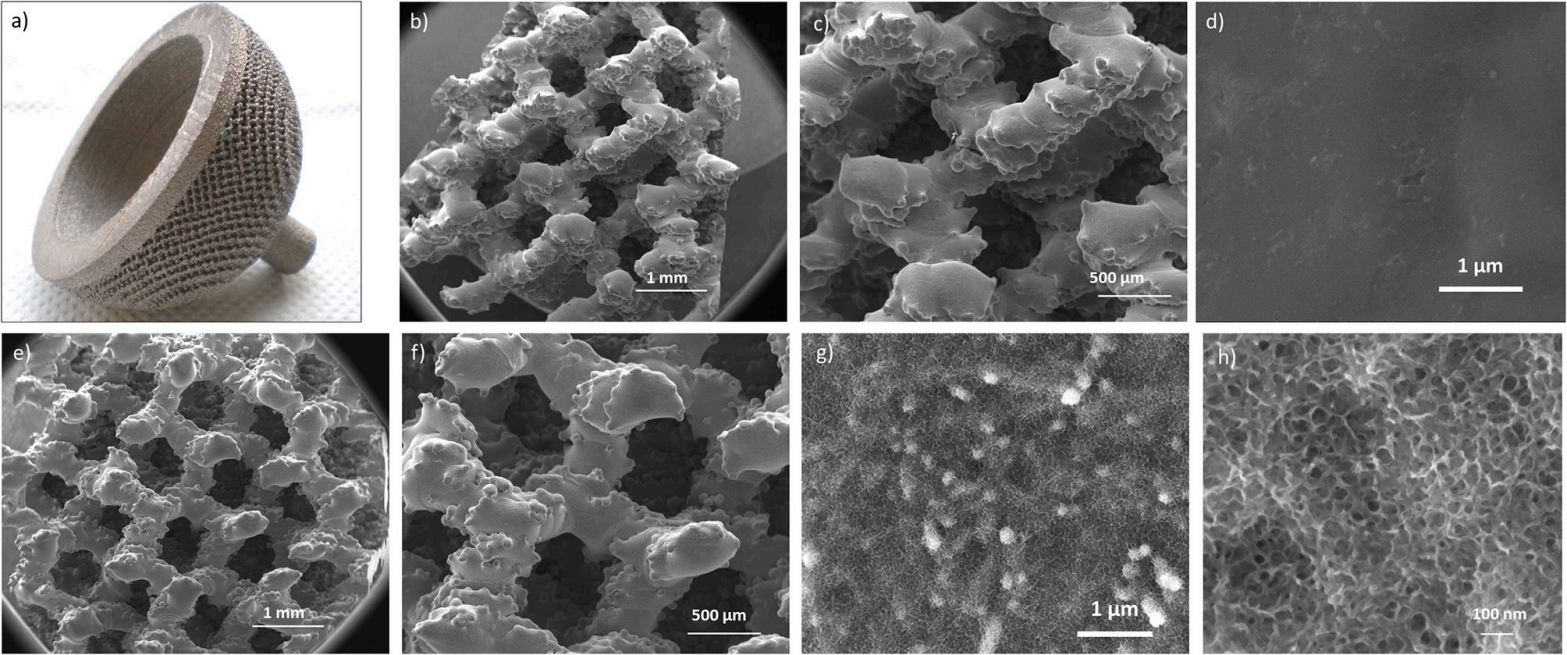
Macroscopic appearance of the untreated cup **(a)**; FESEM images of the untreated **(b–d)** and chemically treated **(e–h)** samples at different magnifications. In the treated specimens the sponge-like nanotexture resulted as homogeneously distributed onto all the cup surface.

An acetabular cup produced through additive manufacturing was used as substrate for the surface chemical treatment as an example of an implant device with a 3D structure at the macroscale. The macroscopic appearance of the considered acetabular cup and the FESEM images of the untreated and surface treated samples obtained from the same device are reported in Figure 3.

The acetabular cup (Figure 3a) has a complex 3D structure, obtained by additive manufacturing (selective electron beam melting), intended for bone integration. Macroscopic interconnected pores are aimed to favor bone ingrowth and mechanical anchoring. The possibility to further improve bone bonding ability by means of a specific surface treatment aimed at overlapping a surface topography with roughness at the nanoscale depends firstly on the ability of the chemical treatment to preserve the early 3D structure of the cup. It can be observed that the chemically treated acetabular cup (Figures 3d,e) maintained the initial 3D architecture (shown in Figures 3b,c) presenting at the same time a well-developed sponge-like nanotexture all over the trabeculae (Figure 3f).

The semi-quantitative chemical composition (EDS) of the untreated and treated cup samples are reported in Table 1. It can be observed that a significant increase in the surface oxygen content can be registered after the chemical treatment as index of the successful surface oxidation. Moreover, surface carbon contaminants were effectively removed by the chemical treatment. This point is particularly important in view of the improvement of bone bonding ability; in fact, it has been reported that carbon contaminations can hamper the interaction between biomaterials (especially titanium) and host cells (Cassinelli et al., 2003; Park et al., 2012; Shi et al., 2016).

**Table 1:** EDS analyses on untreated and treated cup samples.

	**Elements atomic %**	
	**Untreated**	**Treated**
C	16.66	-
O	-	60.51
Al	8.84	3.63
Ti	72.06	34.77
V	2.44	1.10

*Results are expressed as atomic % of each recovered element*.

**Figure 4:**
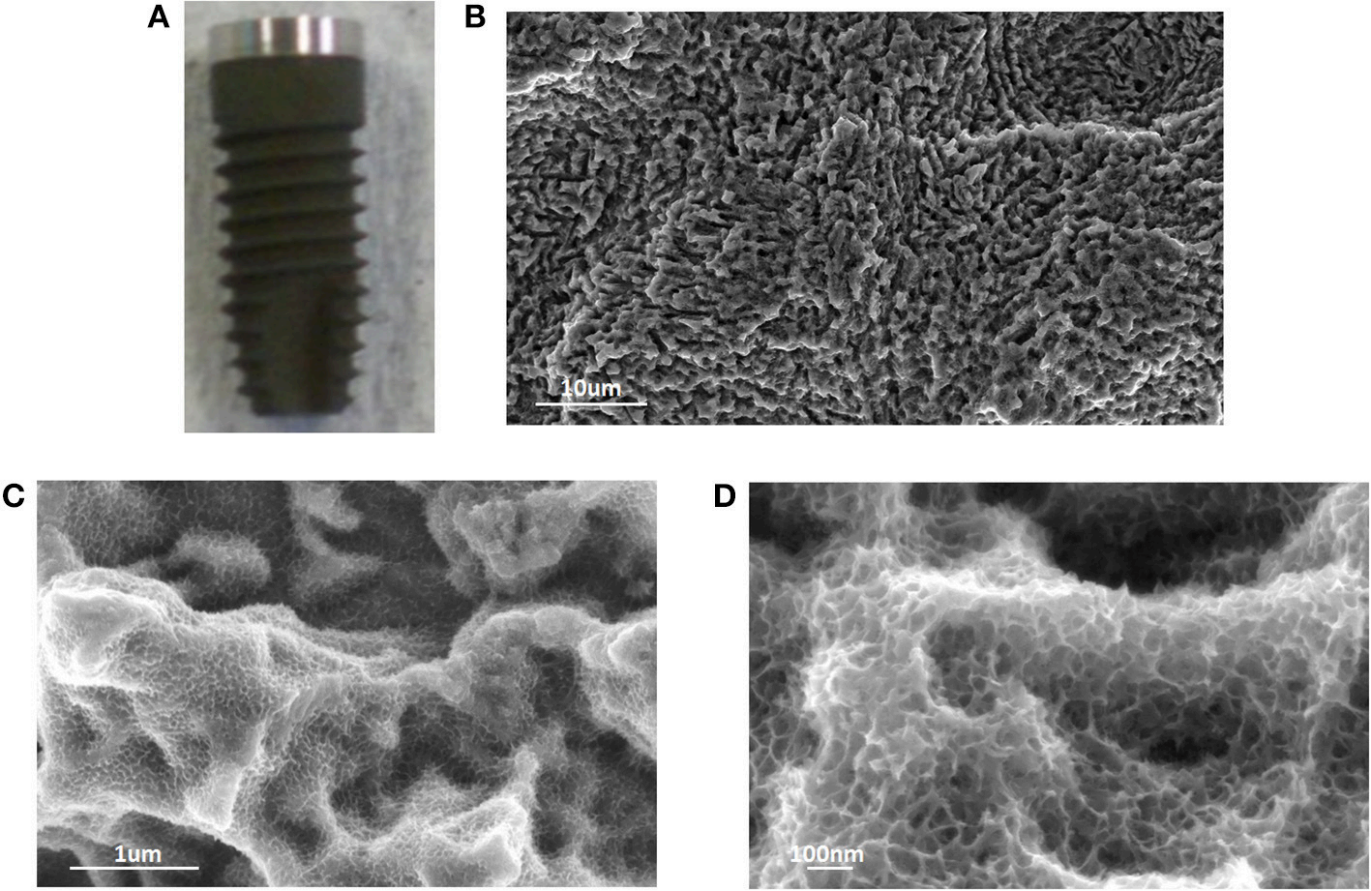
Macroscopic appearance **(A)**, and FESEM observations **(B–D)** of a treated dental screw. Once again, the sponge-like nanotexture introduced by the chemical treatment was evident and homogeneous onto the screw surface.

A dual etched dental screw was used as substrate for the surface chemical treatment as an example of an implant device with a surface profile at the microscale. The macroscopic appearance and the FESEM observations of the chemically treated dental screw are reported in Figure 4.

The proposed chemical treatment introduces a slight darkening of the screw surface due to the growth of the oxide layer. The typical micro-topography of dual acid etched surfaces is completely maintained and the sponge like nanotexture homogeneously covered all the surface following the same geometry.

**Figure 5:**
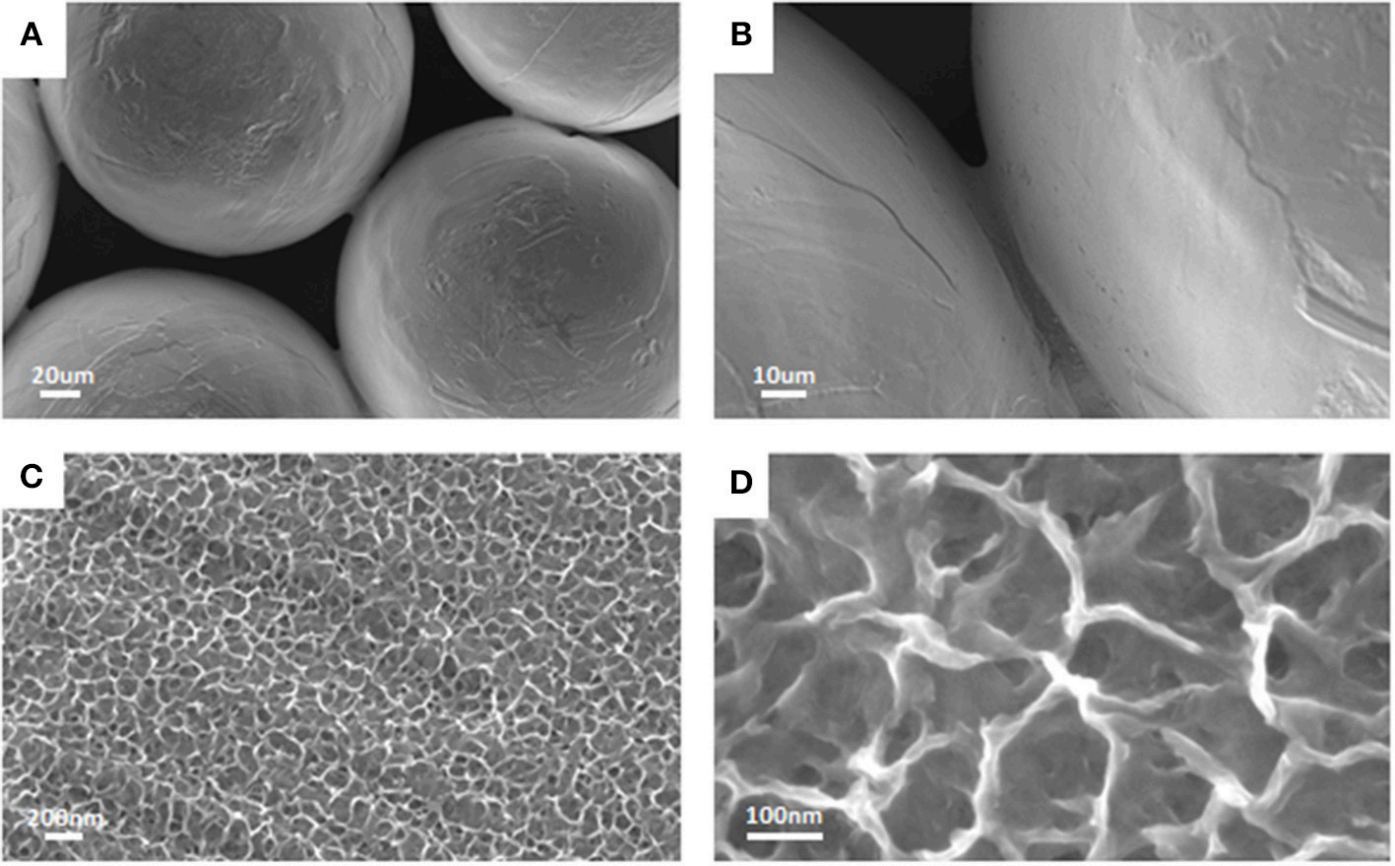
FESEM observations of treated sintered titanium micro-spheres. The original spherical shape was maintained after the chemical treatment **(A,B)** that was effective in introducing the nanotextured layer **(C,D)** as previously observed for cup and screw.

Finally, sintered titanium micro-spheres, representative of laryngeal implants were used as substrates for the surface chemical treatment, as an example of an implant device in contact with soft tissues. FESEM observations of treated titanium micro-spheres are reported in Figure 5. The spherical shape of particles is well-maintained after the chemical treatment, as well as the sintering necks (Figures 5A,B). Moreover, the chemical treatment determined the development of a sponge like nanotexture all over the spheres surface (Figures 5C,D), analogously to what previously observed on the acetabular cup and the dental screw.

The three examples here reported demonstrated that the here proposed chemical treatment can be successfully applied onto commercial three-dimensional devices (e.g., orthopedic, dental and laryngeal implants) within a conventional production cycle, maintaining the starting peculiar topography of the device and adding roughness at the nanoscale, to obtain a fully multi-scale topography (macro or micro and nano) and a unique surface chemistry.

The here discussed chemical treatment requires <3 h to be completed and it is based on the use of solely inorganic reagents. Accordingly, the final products can be sterilized and stored as conventional titanium implants. Moreover, the final surface composition (titanium oxide layer) does not contain any additional element with respect to conventional Ti-based implants thus avoiding any significant complication in the certification processes. So, it can be speculated that all the products obtained by the chemical treatment can fully comply with industrial processes.

### Biological Characterization of the Plane Specimens

#### Evaluation of Biofilm Formation

**Figure 6:**
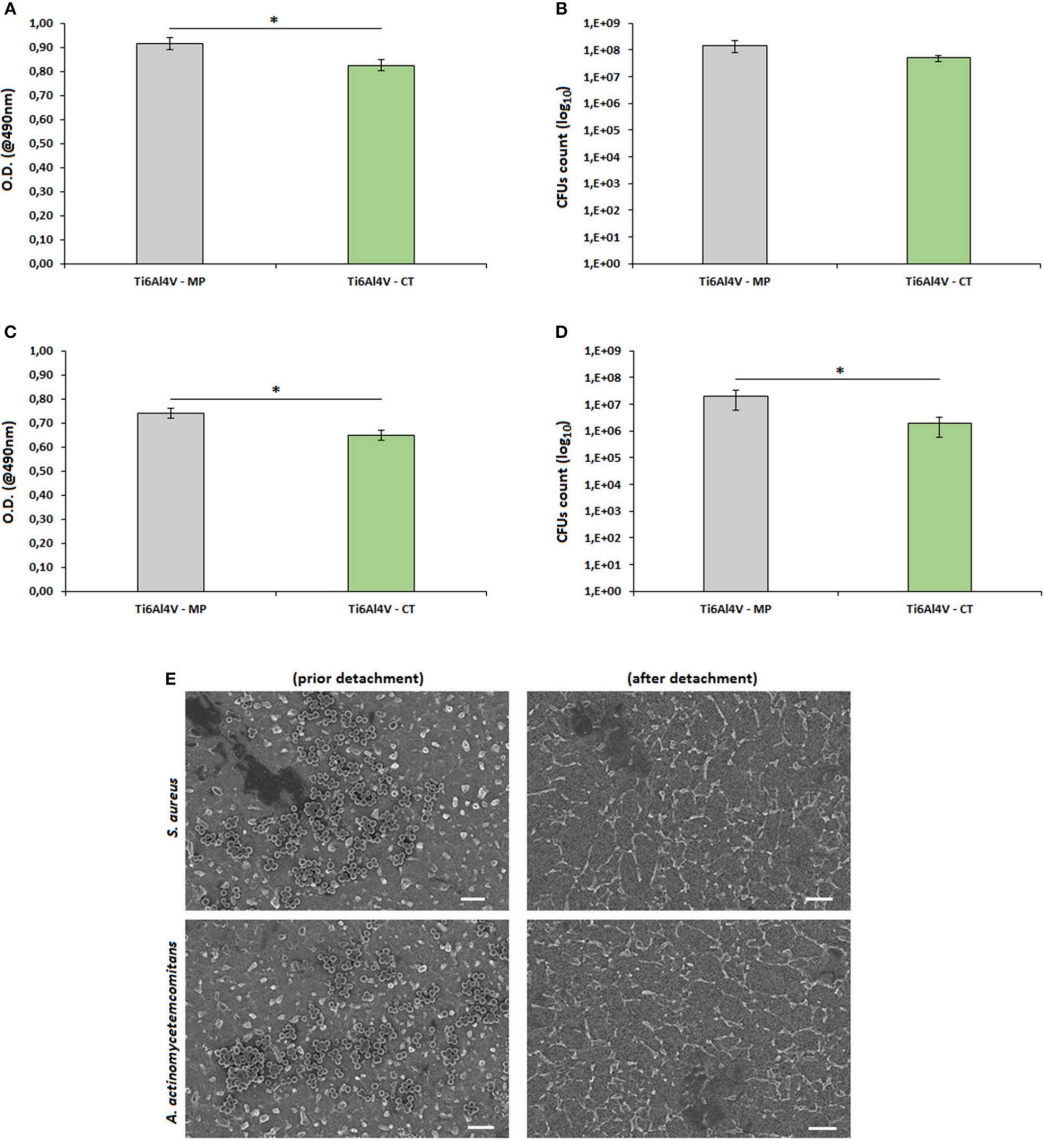
Biofilm adhesion. The surface nanotexture (Ti6Al4V—CT) was able to significantly reduce bacteria metabolism of *S. aureus* (**A**, *p* < 0.05, indicated by^*^) and *A. actinomycetemcomitans* (**C**, *p* < 0.05, indicated by^*^) in comparison with the smooth controls (Ti6Al4V—MP). This reduction was probably due to the lowering number of adhered bacteria as demonstrated by the reduction of viable colonies count (CFU) of *S. aureus* (**B**, *p* < 0.05, indicated by ^*^) and *A. actinomycetemcomitans* (**D**, *p* > 0.05), respectively. Bars represent means and standard deviations. SEM images (**E**, bar scale = 2 μm, magnification = 10,000x) confirmed that bacteria were successfully detached from specimen's surface and that the detected differences were due to surfaces' properties.

The results concerning biofilm formation on the treated and untreated surfaces are reported in Figure 6.

In general, the nanotexture introduction onto the Ti surface determined an increase of the specimens' anti-adhesive properties; in fact, a significant reduction in terms of bacterial metabolism was observed for both *S. aureus* (a, *p* < 0.05, indicated by the ^*^) and *A. actinomyectemcomitans* (c, *p* < 0.05, indicated by the ^*^). So, as a first reading of these results, the effect seems to be not strain-related as similar values were obtained for 2 different strains. A similar anti-adhesive effect due to a nanoporous surface modification was previously described also by Narendrakumar et al. (2015): they demonstrated that the adhesion of *Streptococcus sanguinis* and *Streptococcus mutans* onto TiO_2_ nanoporous surfaces was strongly decreased in comparison with the same untreated or nanotubes-doped surfaces. More recently, it was further demonstrated that the introduction of a nanofiber structure onto a Ti surface was able to significantly decrease the adhesion of *Streptococcus mutans* in comparison with the same smooth or microfiber-doped materials (Miao et al., 2017).

As prior mentioned, most of the literature deals with the hypothesis that the reduction of bacterial metabolism is due to their difficulty to adhere onto the nanotextured surfaces. Accordingly, we performed a CFU count and we confirmed that the number of viable colonies adhered to the specimens' surface was lower for the Ti6Al4V—CT treated samples in comparison to the TI6Al4V—MP control ones for both *S. aureus* (b, *p* > 0.05) and *A. actinomytemcomitans* (d, *p* < 0.05, indicated by the ^*^). So, it was possible to correlate the reduction of biofilm metabolism with the lowering of viable adhered colonies, thus confirming what supposed from previous literature.

As a further verification of the obtained results, SEM images (e) demonstrated that all adhered bacteria were successfully detached by vortex and sonication, thus excluding possible results differences due to experimental procedure limitations.

In fact, considering that no antibiotics or active molecules were grafted onto the surface, the observed antiadhesive effect is obviously related to the surface nanotexture. If compared to eukaryotic cells, bacteria hold a less elastic membrane and a higher stability in terms of shape maintenance; so, they result as much more sensitive to surface irregularities. The effect of such surface modifications has been shown to strongly affect bacteria adhesion and subsequent functions when they are realized in the nano-scale, that is the same size of bacteria (Puckett et al., 2010; Izquierdo-Barba et al., 2015). The presence of these surface nanotextures represents a strong limitation for bacteria adhesion as they strongly restrict the availability of berth points; so, this limited free-area impairs the adhesion of high-density bacteria (Bagherifard et al., 2015).

Based on these considerations and to the here obtained results, nanotextured surfaces appears an interesting and emerging strategy to counteract metal devices infection coupled with other approaches. Moreover, the here proposed strategy does not induce the release of active antibacterial agents overcoming the risks related to cytotoxicity as well as the complication in the medical device classification. However, it must be considered that the present results are limited to a preliminary *in vitro* evaluation of this novel surface treatment and that they are not yet satisfactory for a clinical point of view. In general, the success of this strategy could be affected by 2 main problems. The first one is represented by the temporal length of the bacterial activity. Whereas surface nanotexturing was able to satisfactorily limit the early stage of bacteria adhesion, it failed to inhibit long term colonization (Ribeiro et al., 2012). The second issue is related to the nature of bacteria themselves. Gram-positive bacteria hold a rigid peptidoglycan layer which does not allow fluidic movements: accordingly, they are not able to adapt to the nanotextured topography, thus failing surface adhesion. On the opposite, Gram-negative bacteria are favorites by the presence of an extra outer membrane that allows to a more fluidic interaction with the nano-irregularities of the treated surface, thus allowing the anchorage. In this work, we obtained comparable results for both Gram-positive (*S. aureus*) and Gram-negative (*A. actinomycetemcomitans*) bacteria but, as an example, Bagherifard et al. (2015) obtained positive response with *S. aureus* and *S. epidermidis* (both Gram-positive) but a bad outcome from *P. aeruginosa* (Gram-negative).

#### Cytocompatibility

This test was performed to evaluate the cytocompatibility of the Ti6Al4V- CT samples despite of their ability to reduce bacterial contamination.

**Figure 7:**
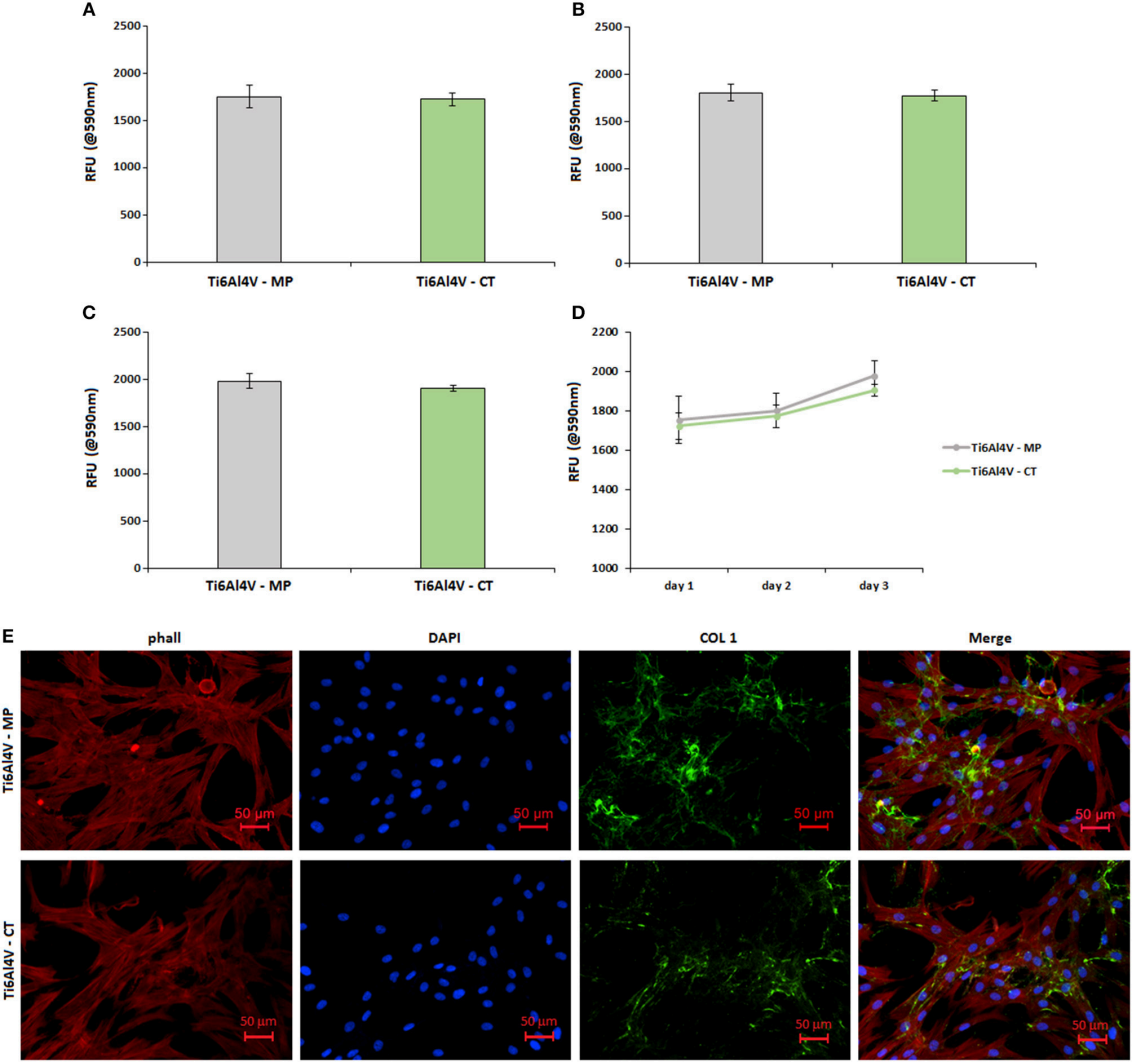
Cytocompatibility evaluation. No differences were noticed in terms of cells viability between the untreated control (Ti6Al4V—MP) and the treated specimens (Ti6Al4V—CT) after 1 **(A)**, 2 **(B)**, and 3 **(C)** days of direct cultivation; result were comparable and not significant (*p* > 0.05) as summarized in **(D)**. Moreover, cells were able to deposit matrix onto both surfaces as demonstrated by the evident collagen I (COL 1) accumulation after 3 days **(E)**. Bars represent means and standard deviations.

The viability results obtained after 1-2-3 days in direct contact within hFOB cells and specimens' surface are summarized in Figure 7. No differences were noticed between Alamar™ starting solution and the materials solely (intended as cells-free) thus excluding any unwanted reding background due to the presence of active species on the specimens' surface.

No significant differences (*p* > 0.05) were noticed by comparing the Ti6Al4V—MP control specimens with the treated Ti6Al4V—CT ones. In fact, optical density values were very similar at each time points of 1 (a), 2 (b), and 3 (c) days; moreover, the obtained values showed a similar increase during the 3 days of direct culture, thus showing a comparable cells ability to proliferate onto the untreated control surface and the nanotextured one (as summarized in d). So, the here proposed nanotexture showed to be cells-friendly toward human osteoblasts progenitor cells.

Fluorescence images (Figure 7E) were useful to confirm that seeded cells were able to correctly adhere and spread onto both control Ti6Al4V—MP and treated Ti6Al4V—CT ones. In fact, phalloidin staining (E, stained in red) revealed a correct cytoskeleton conformation, as well as nuclear DAPI staining (e, in blue) confirmed that a comparable number of cells were adhered to the treated surfaces in comparison with the control ones. Moreover, looking at the cell's matrix deposition by collagen I fluorescent staining (e, stained in green), it was possible to verify that cells were able to undergo extracellular matrix deposition onto the nanotextured surface in a comparable manner with untreated control.

This short cellular test is not able to show a promotion on osteoblast differentiation, but asses the cytocompatibility of the treatment. A significant increase in osteoblasts proliferation and differentiation on the nanotextured titanium surfaces was previously observed by the Authors through different cellular cultures (Spriano et al., 2013). This is in agreement with literature. Malec et al. (2016) demonstrated that the presence of a nanoporous anodic titanium oxide layer onto bare grade II titanium was effective not only in support adipose derived stem cells proliferation, but even to promote osteogenesis due to the nanotexture feature.

Moreover, besides its pro-osteointegrative role, the nanotextured layer can be also useful as platform for further improvements: as an example, Wu et al. (2013) functionalized a microporous titanium oxide layer through the lyophilization of microRNA that were aimed to enhance osteogenesis through the miR-29b pathway. Similarly, the Authors successfully employ surface hydroxyl groups for the grafting of alkaline phosphatase (Ferraris et al., 2011b).

## Conclusion

Despite the large use of Ti and its alloys in orthopedic and dentistry, the clinical success of such devices is still limited by the long-term lack of osseointegration in critical patients' situations and the surface colonization of drug resistant pathogens. Accordingly, there is an urgent need to introduce pro-osseointegrative and antibacterial treatments to improve devices clinical success.

Here we introduced a nanotextured titanium oxide layer that demonstrated to match with the above-mentioned requirements, showing very promising results; moreover, the technology used for the surface treatment was successfully scaled to real medical devices thus filling the gap between the “bench” and the real scenario application.

## Author Contributions

SF, MC, AC and AS performed the experiments, discussed the results and wrote the first draft of the work. MT was responsible for AFM and FESEM analysis. SS and LR coordinated the work, discussed the results, participated in the first draft of the paper, and performed its final revision.

### Conflict of Interest Statement

The authors declare that the research was conducted in the absence of any commercial or financial relationships that could be construed as a potential conflict of interest.
